# Opioid-Based Haptens: Development of Immunotherapy

**DOI:** 10.3390/ijms25147781

**Published:** 2024-07-16

**Authors:** Sándor Hosztafi, Anna Rita Galambos, István Köteles, Dávid Á Karádi, Susanna Fürst, Mahmoud Al-Khrasani

**Affiliations:** 1 Department of Pharmaceutical Chemistry, Semmelweis University, Hogyes Endre u. 9., H-1092 Budapest, Hungary; istvan.koteles@gu.se; 2 Department of Pharmacology and Pharmacotherapy, Faculty of Medicine, Semmelweis University, Nagyvá-rad tér 4., H-1445 Budapest, Hungary; galambos.anna@phd.semmelweis.hu (A.R.G.); karadi.david.arpad@semmelweis.hu (D.Á.K.); furst.zsuzsanna@med.semmelweis-univ.hu (S.F.); 3 Department of Chemistry and Molecular Biology, University of Gothenburg, SE-412 96 Gothenburg, Sweden; 4 Department of Anesthesiology and Intensive Therapy, Semmelweis University, Üllői út 78., H-1082 Budapest, Hungary

**Keywords:** morphine, heroin, oxycodone, fentanyl, opioid, hapten, vaccination, immunotherapy

## Abstract

Over the past decades, extensive preclinical research has been conducted to develop vaccinations to protect against substance use disorder caused by opioids, nicotine, cocaine, and designer drugs. Morphine or fentanyl derivatives are small molecules, and these compounds are not immunogenic, but when conjugated as haptens to a carrier protein will elicit the production of antibodies capable of reacting specifically with the unconjugated hapten or its parent compound. The position of the attachment in opioid haptens to the carrier protein will influence the specificity of the antiserum produced in immunized animals with the hapten–carrier conjugate. Immunoassays for the determination of opioid drugs are based on the ability of drugs to inhibit the reaction between drug-specific antibodies and the corresponding drug–carrier conjugate or the corresponding labelled hapten. Pharmacological studies of the hapten–carrier conjugates resulted in the development of vaccines for treating opioid use disorders (OUDs). Immunotherapy for opioid addiction includes the induction of anti-drug vaccines which are composed of a hapten, a carrier protein, and adjuvants. In this review we survey the design of opioid haptens, the development of the opioid radioimmunoassay, and the results of immunotherapy for OUDs.

## 1. Introduction

Opioid ligands alone do not possess immunogenic properties, but coupled with a protein carrier they can elicit an immune response, and the formation of a specific type of antibodies. This observation is very important from a pharmacological point of view because immunotherapy is based on these immunogen formulation techniques.

**Figure 1:**
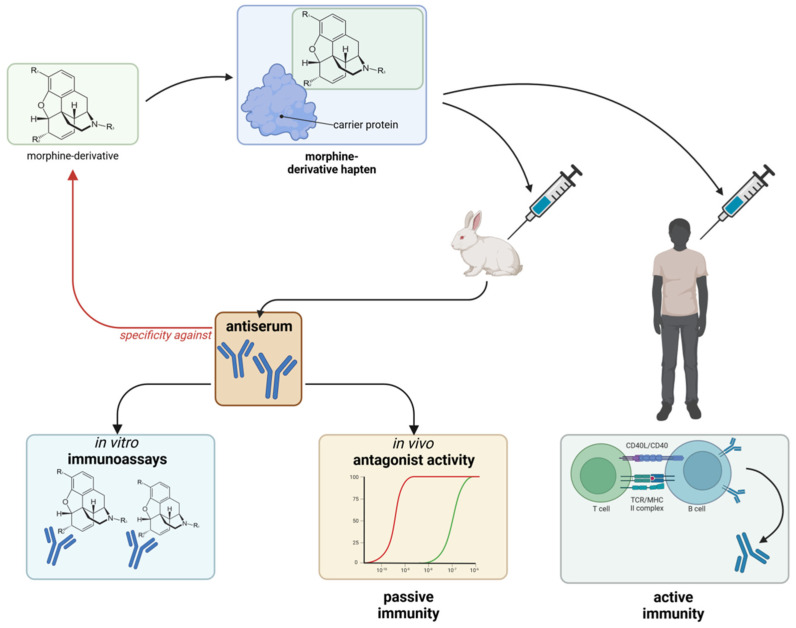
Morphine-based immunisation or vaccination proposed for the treatment of OUD.

A hapten may be defined as a low molecular weight substance too small to be immunogenic by itself, but when covalently coupled to a large immunogenic protein carrier in the presence of a proper adjuvant and injected into an animal will give rise to the formation of antibodies specific for the chemically coupled small molecule (Figure 1). The reactive carboxylic or amino group in a hapten could be used to conjugate it to the protein molecule. The carrier proteins that are usually employed in the preparation of hapten–protein conjugates are bovine serum albumin (BSA), keyhole limpet hemocyanin (KLH), and tetanus toxoid (TT). These types of proteins are being used in the formulation of immunogens in preclinical studies involving drugs of abuse [1,2,3]. BSA, a 66 kDa protein, is an attractive carrier for vaccine development because it contains 30–35 surface lysines accessible for conjugation and it has high aqueous solubility. This protein is commercially available in acceptable purity, however BSA is not suitable for human vaccines in the United States or Europe due to the risk of prions. On the other hand, KLH has been extensively studied in human clinical trials as a carrier for therapeutic cancer vaccines yet contains ~2000 lysines allowing for the easy conjugation of haptens. It is important to consider that the antigen recognition and processing system of an animal is stimulated only by the injection of large molecules such as hapten–protein conjugates, and antibodies formed in response to this stimulation recognize and bind only to small segments called antigenic determinants of the injected hapten–protein conjugate (Figure 1).

In the last three decades, the opioid crisis has created a significant social and economic burden. The opioid epidemic crisis has stemmed from repeated exposure to opioid agonists acting on µ-opioid receptors (MORs) and can occur in individuals who use opioids for either pain relief or non-medical purposes [4,5,6,7,8]. In the late 1990s, the increasing rates of opioid use and misuse led to an epidemic of abuse of opioid prescriptions. The over-prescription of opioid analgesics such as oxycodone (OxyContin) resulted in a high rate of abuse and diversion. The rise in prescription opioid-related overdose deaths slowed in 2010, a year when a long-acting oxycodone formulation was introduced into the market [9,10]. This change in the formulation makes it harder to insufflate or inject. In the same year, an abuse-deterrent formulation of OxyContin was developed, and oxycodone addicts switched to heroin because their dependence required another drug. In the 2010s, the United States and western Canada witnessed a dramatic increase in heroin overdose rates, during which rates more than tripled. Since heroin was readily available and less expensive than oxycodone, users became heroin addicts [11]. Initially, heroin was adulterated with fentanyl, but in 2016, fentanyl became a surrogate for heroin. This is because fentanyl displays an advantage over heroin in relation to its potency; a small amount is needed to achieve a euphoric effect, and the small size and weight make it easy to transport and distribute. In addition, the low cost and availability of starting material for clandestine synthesis are also largely attributed to the illicit drug trade of fentanyl [12]. The illegally manufactured fentanyl, its analogues, and other novel synthetic derivatives entering the illicit market alone or as a mixture of heroin have resulted in a dramatic increase in overdose deaths in North America and, to a certain extent, in Europe [8,13]. According to recent economic studies, a shocking 136 opioid-related overdose deaths occur on a daily basis [14]. All of these recent dramatic changes in OUDs have encouraged researchers in the last decades to develop vaccines against natural and semisynthetic opioid alkaloids as well as synthetic opioids, such as fentanyl-based opioid structures, which are discussed in this review.

## 2. The Background of Opioid-Based Hapten Structures 

**Figure 2:**
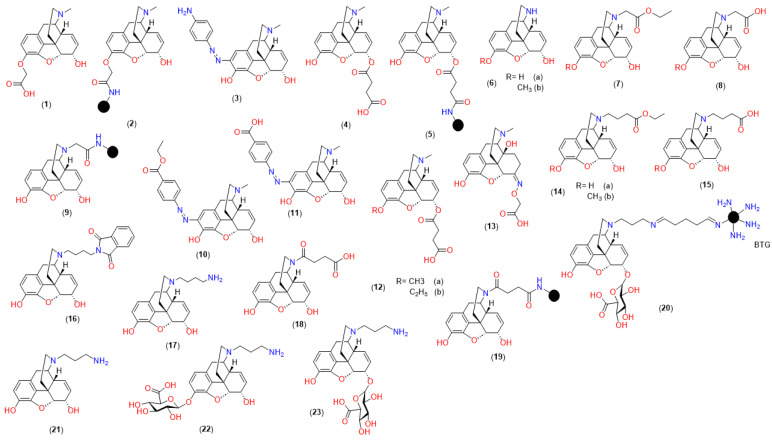
Structures of morphine-based haptens and immunoconjugates (2, 5, 19 and 20).

**Figure 3:**
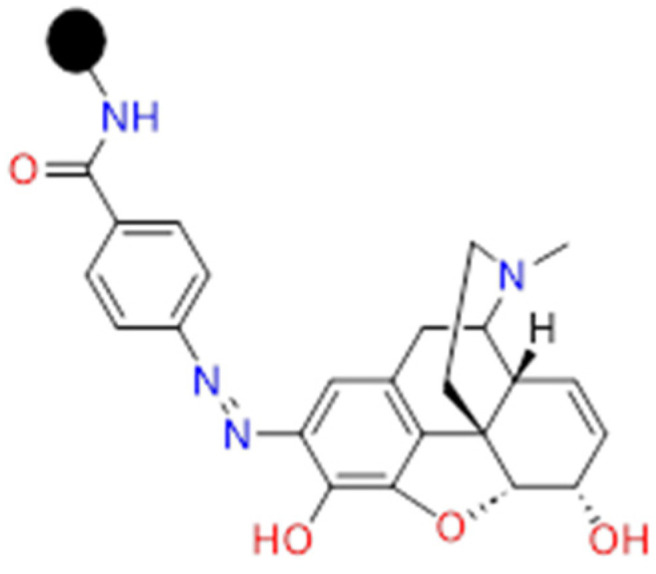
The bioconjugate structure of 2-arylazomorphine-BSA, adopted from Catlin et al. [24].

A large number of morphine-based structures (Figure 2) have been developed by several research groups throughout the past decades that have opioid-hapten properties [1,15,16,17,18,19,20,21,22,23,24,25,26,27,28,29,30,31,32,33,34,35,36,37,38,39,40,41,42,43,44,45,46]. Some of these structures were conjugated to a carrier protein in either the C-2, C-3, C-6, or N-17 position (Figure 2 and Figure 3). The formed conjugates were mixed with Freund’s adjuvant to form an emulsion for injection. Subsequently, animals were immunized with the produced vaccines to induce the production of antibodies [1,15,16,17,18,19,20,21,22,23,24,25,26,27,28,29,30,31,32,33,34,35,36,37,38,39,40,41,42,43,44,45,46]. The immune specificity and cross-reactivity of the prepared vaccines are depicted in Table 1. Indeed, the opioid-based hapten structures were first developed by Spector and co-workers [15,16] in the seventies of the last century with the aim of determining the morphine in blood or urine by radioimmunoassay (RIA). In this work, morphine base was converted to 3-*O*-carboxymethylmorphine (1) in a reaction with the sodium salt of chloroacetic acid. This compound was coupled to BSA in an aqueous solution in the presence of 1-ethyl-3(3-dimethylaminopropyl)-carbodiimide as a coupling agent. The conjugate (2) was dialyzed against water. Rabbits were immunized with this conjugate, emulsified with Freund’s adjuvant. Antiserum was collected from the blood and subjected to RIA. Antiserum was incubated in the presence of tritium-labelled dihydromorphine and the radioactivity of antibody-bound morphine was determined. The addition of unlabelled morphine to a fixed amount of ^3^H-dihydromorphine and antiserum resulted in a competitive inhibition of labelled dihydromorphine for the formation of the antibody–hapten complex. An extremely low concentration of morphine (0.5 ng) can be measured by this assay [1,15]. Furthermore, Spector et al. [17] prepared another hapten of morphine in the reaction of the diazonium salt of *p*-amino-acetanilide and morphine. After *N*-deacetylation 2-(*p*-aminofenilazo)-morphine (3) was also coupled to BSA, and this conjugate elicited the formation of antibodies. The antiserum was incubated with ^3^H-labelled-morphine and the inhibition concentrations of opioids were determined. The concentration of morphine which inhibited the antigen–antibody complex in 50% proved to be 0.75 ng. For codeine and heroin, the same values were 0.9 ng and 2.3 ng, respectively. Wainer et al. [18,19] synthesized a hemisuccinic acid ester of morphine which conjugates to BSA forming stable, easily definable hapten–protein conjugates. Morphine-6-hemisuccinate (4) was prepared by heating a mixture of morphine with three equivalents of succinic anhydride in pyridine for 4 h. The morphine-6-hemisuccinate was conjugated to BSA by the mixed anhydride method. The reaction of morphine-6-hemisuccinate with isobutyl chloroformate was performed in dioxan solvent in the presence of tributylamine. BSA was added to the mixed anhydride and after 4 h of stirring the reaction mixture was concentrated by pressure dialysis against water. Eleven New Zealand white rabbits were inoculated subcutaneously with morphine-6-hemisuccinate-BSA conjugate (5) emulsified in complete Freund’s adjuvant (CFA). The affinity of antibodies was determined, and the specificity of antigen binding was demonstrated by competition studies with the addition of unlabelled morphine or other opiates prior to that of 14C-labeled morphine. Drug concentrations which inhibited 50% of the binding of 14C-morphine (I50) were as follows: morphine 110 pmol/mL; heroin 100 pmol/mL; and codeine 160 pmol/mL. Both morphine-6-hemisuccinate and 3-*O*-carboxymethyl-morphine are assumed to form amide linkages with free amino groups on the BSA molecule. Gintzler et al. [20] prepared an N-substituted-normorphine hapten and this hapten was coupled to BSA. Normorphine (6a R=H) was N-alkylated with the ethyl ester of bromoacetic acid in dimethyl formamide solvent. The ester group of *N*-ethoxycarbonylmethyl-normorphine (7) was hydrolysed by heating with 2N HCl. The free carboxyl group of *N*-carboxymethyl-normorphine (8) was coupled to BSA in aqueous solution in the presence of 1-ethyl-3(3-dimethylaminopropyl)-carbodiimide. The sensitivity of the RIA for morphine using the *N*-carboxymethyl-normorphine based antiserum (9) was determined. The addition of an increasing amount of unlabelled morphine to a fixed amount of ^3^H-dihydromorphine and antiserum results in a competitive inhibition of the labelled antibody–hapten complex. It was found that 30 pg of unlabelled morphine was sufficient to produce a 50% displacement of labelled dihydromorphine. The degree of binding of various opiates to the antibody was measured by incubating the opioids with the antibody in the presence of labelled dihydromorphine and measuring the degree of inhibition of the labelled hapten–antibody complex. After this short historical view on the development of opioid haptens, the review will focus on the preclinical studies of immunotherapy in the scenario of OUDs. 

## 3. Pharmacological Studies on Anti-Morphine Antisera

Berkowitz and Spector [52] reported on the production of antibodies which bind morphine in mice that have been actively immunized with a conjugate of morphine and protein, yet an altered effect and biologic disposition of morphine in these mice were observed. The morphine immunogen, 3-carboxymethylmorphine coupled to BSA, was previously shown to be effective in producing antibodies specific for morphine in the rabbit. Mice were also able to develop antibodies against morphine. Mice were injected subcutaneously once a week for 16 weeks with 1 µg of morphine immunogen in 50 percent CFA emulsion. The sera from mice injected with the morphine immunogen in CFA bound ^3^H-dihydromorphine. The binding of the dihydromorphine was specific, and morphine could displace ^3^H-dihydromorphine from the antibody. The in vitro binding of dihydromorphine suggests the presence of antibodies that can bind narcotics in actively immunized mice. Additional evidence supporting this hypothesis was obtained in an in vivo study. Mice were immunized for 16 weeks, or were injected with saline or adjuvant, and then were injected intravenously with 50 µg of ^3^H-dihydromorphine. The mice were killed 1 or 5 h later, and their sera were analysed for dihydromorphine. Mice immunized with the morphine immunogen had 20 times more dihydromorphine present in their serum after 1 h than mice injected with saline (control mice) and had more than 50 times more dihydromorphine after 5 h than did control mice. The data from the in vivo and in vitro experiments show that the serum from mice actively immunized with a morphine immunogen can bind dihydromorphine to a greater extent than the serum of control mice. Morphine analgesia in mice was determined by measuring the ability of morphine to decrease writhing induced by *p*-phenylquinone. The pharmacologic effect of morphine was diminished in mice which had been injected with morphine immunogen in Freund’s adjuvant for 6 or 16 weeks.

Berkowitz et al. [53] examined the influence of active and passive immunization on the disposition of ^3^H-dihydromorphine in mice and rats. The simplest method of immunization was to passively transfer antibodies against morphine obtained from actively immunized rabbits. Rabbits immunized with the morphine antigen 3-*O*-carboxymethyl-morphine coupled to BSA served as the source of the antibodies which were used for the passive immunization of mice and rats. Mice were also actively immunized with the morphine antigen. The 45 min plasma concentration of the ^3^H-dihydromorphine was increased 90–100 fold in passively immunized mice whereas the brain concentration decreased by at least 75 percent. The plasma half-life was markedly slowed in immunized mice. The authors suggested that the antibodies may initially act to sequester the opioid but over time, as the narcotic is slowly released from the antibodies, they may also act as a circulating source of the drug. It is apparent that the presence of circulating antibodies can have marked effects on the disposition of narcotics.

Wainer et al. [54] performed a study to determine if antibodies reactive with morphine could also inhibit or reverse one of the pharmacological actions of the drug. The research team examined the capacity of rabbit antibodies produced by immunization with morphine-6-hemisuccinate-BSA to antagonize the depressant action of morphine on electrically induced contractions of isolated guinea pig ileum (GPI). A concentrated crude globulin fraction of hyperimmune serum obtained from the immunized rabbit was prepared and purified by pressure dialysis. The assay for morphine activity was based on the inhibitory action of morphine on the electrically stimulated contractions of the longitudinal muscle of GPI. The inhibitory effect of increasing concentrations of morphine hydrochloride on the muscle twitch was determined and 70–80% inhibition was achieved with 60 nM morphine. The capacity of anti-morphine-6-hemisuccinate-BSA to antagonize the depressant actions of morphine on the ileum was measured. Varying morphine concentrations were preincubated with antibody globulin for 15 min at room temperature, and then added to the organ bath for 3 min. This amount of antibody inhibited the effect of morphine concentrations ranging from 60 nM to 1000 nM. Morphine preincubated with normal rabbit serum globulin retained full activity, and normal rabbit serum globulin alone had no impact on the GPI muscle contraction. This prevention of the pharmacological effect of morphine on isolated GPI was probably due to the morphine–antibody combination stopping the morphine from reaching the receptor sites. The prevention of the morphine effect was complete when a two-fold stoichiometric excess of antibodies was present. To determine if the antibody was able to reverse morphine action, antibody was added to the organ bath 1 min after the addition of morphine when the induced inhibition of muscle contraction was maximal. Anti-morphine-6-hemisuccinate-BSA added for 1 min after morphine completely reversed the inhibitory effect of 120 nM morphine on muscle contraction. Normal rabbit serum globulin had no effect on the morphine-induced inhibition of the muscle contraction.

The results of these experiments indicate that specific antibodies are capable of antagonizing the inhibitory effect of morphine on the electrically stimulated contractions of GPI. The antibody molecules apparently bind morphine more strongly than the tissue receptor site as antibodies were able to prevent and reverse the inhibitory effect of morphine on smooth muscle contraction.

**Scheme 1:**
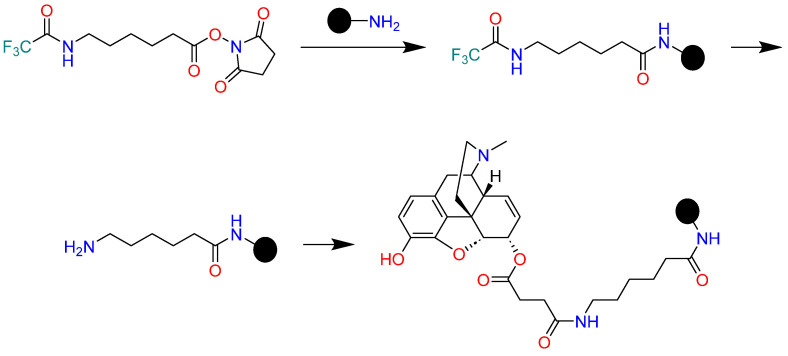
Synthesis of morphine-6-hemisuccinyl-bovine serum albumin conjugate.

In an in vivo study, Bonese et al. [2] investigated the effect of immunization against an opiate on the self-administration of these drugs in a rhesus monkey. The animal was trained to self-administer heroin and cocaine in saline solutions. Cocaine was used to detect nonspecific changes in drug-taking behaviour after immunization. One of the two drugs was available each day for a 2 hr session, and the sequence of drug availability was varied. The appearance of a stimulus light overhead and another on the appropriate lever signalled the start of each drug session. After heroin and cocaine intake had stabilized, the substitution of normal saline demonstrated that the self-administration of heroin and cocaine were independent of each other and that the monkey could detect the absence of either drug despite the presence of the usual stimuli associated with drug reinforcement. After a two-months period for which data show that tolerance was not a significant factor in regulating self-administration, the animal was immunized subcutaneously with 5 mg morphine-6-hemisuccinyl-BSA conjugate (Scheme 1). A second 5 mg dose was given 28 days later, and every 2 weeks thereafter, a 10 mg dose was given for a total of 20 weeks. Results indicate that *Rhesus* monkeys can be induced to produce antibodies against opiates. The anti-opiate immunoglobulin G was present in both serum and cerebrospinal fluid. The blockade of CNS actions of heroin by this antibody was also demonstrated. Killian et al. [55] studied the effects of anti-morphine antibodies produced in *Rhesus* monkeys immunized with morphine-6-hemisuccinate-BSA that were passively administered to recipient monkeys trained to self-administer heroin and cocaine. The results of these experiments confirmed that the passive transfer of anti-morphine antibodies resulted in a significant increase in the amount of heroin self-administration by conditioned monkeys.

Anton et al. [48,49] utilized new concepts for the design of heroin/morphine—carrier protein conjugates in order to induce specific antibodies. Since BSA- and KLH-based morphine or heroin vaccines could not be used in clinical trials because such protein carriers are not licensed for human use, Anton et al. developed a novel model of a morphine vaccine, based on the coupling of a morphine-6-hemisuccinate derivative to TT, and this carrier protein is highly immunogenic. The linking part between the hapten and the carrier is the linker, which highlights the characteristic structure of the hapten on the surface of the antigen to elicit more specific antibodies, and it was experienced that the linker should be far from the functional groups to avoid affecting the recognition of the antibody by the hapten. The length of the linker and its degree of freedom can help to place the hapten–carrier protein conjugate in an appropriate spatial orientation on the protein surface to be recognized as a potential dominant epitope antigen. Therefore, the drug hapten-linker arm should be covalently attached to reactive chemical groups exposed in the protein carrier, which usually are the epsilon (ε) amino groups in either lysine or arginine residues. In an inspection of the models, Anton et al. compared the length of the linkers in three opioid hapten protein conjugates. In the 3-*O*-carboxymethyl-morphine, which was coupled to the (ε)-amino groups of the lysine residue of the BSA carrier, the length of the linker was found to be ≈8.96 Å. In the coupling of morphine-6-hemisuccinate to the (ε)-amino group of the lysine residue of the BSA carrier, the length of the linker was ≈12.41 Å. In the case of the morphine-6-hemisuccinate—TT conjugate a larger length of the linker was found, at ≈20.15 Å.

It has been demonstrated that both the length of the linker and degrees of freedom place the haptenized moieties of each conjugate distant enough from the structural domains of the carrier protein and in an appropriate spatial orientation. This longer linker, through which morphine is covalently linked to the hapten carrier protein, was utilized to stabilize the amide bonds.

The linker was prepared by the reaction of ε-(*N*-trifluoracetylamino-caproic acid *N*-hydroxysuccinimide ester) with the amino group of the lysine residue of TT. Then the trifluoracetyl protecting group was removed at pH = 8.1. This free amino group of the TT conjugate was acylated with the carboxyl group of morphine-6-hemisuccinate using 1-ethyl-3-(3-dimethylaminopropyl) carbodiimide hydrochloride (EDC) as a coupling reagent (Scheme 1).

In these drug vaccine models, the central goal is to present the covalently attached drug as the dominant epitope determinant to immune system antigen-presenting cells to elicit an immune response for the formation of high levels of specific anti-drug circulating antibodies. Since antibodies are immunoglobulin proteins having very large molecular mass (≈150 kDa), they cannot readily cross the blood–brain barrier and enter the brain’s tissue. Therefore, any drug that is bound to the antibody molecule also could not cross the blood–brain barrier and cannot enter the brain. In this context, the medical rationale for using anti-drug antibodies in the serum as a treatment is to reduce drug levels in the brain and to bind the drug before entering the brain. 

A competition ELISA was used to evaluate the specificity of the anti-morphine antibodies. The antisera recognized with equivalent specificity 6-*O*-acetylmorphine, M3G, and M6G. These data suggest the immunogenic capability of the morphine-6-hemisuccinate–TT vaccine to generate a humoral response of anti-morphine antibodies displaying equivalent specificities not only for heroin but also for its active metabolites. The research team studied morphine-6-hemisuccinate—TT efficacy in preventing relapse to morphine addictive self-administering behaviour in the same animal model. These experiments used the rat model of the relapse of intravenous (iv.) self-administration of either morphine or heroin, where groups of animals were trained to self-administer a moderate reinforcing dose of either morphine (600 μg/kg) or heroin (60 μg/kg). After attaining stable drug-intake responses, trained animals were subjected to a period of drug extinction by substituting the drug with saline. During this period, animals were vaccinated with at least 4–6 boosts with the morphine-6-hemisuccinate—TT vaccine, and then re-challenged with the same unit dose of either morphine or heroin. The monitoring of humoral immune response showed high serum antibody levels in vaccinated animals. Conversely, to control animals vaccinated with either adjuvant alone (aluminium hydroxide gel) or TT plus aluminium gel, immunized animals with the morphine-6-hemisuccinate—TT vaccine did not reacquire either morphine- or heroin-taking behaviour throughout the 15 days of daily testing with the same morphine or heroin addictive unit doses. For the group of subjects immunized with the morphine-6-hemisuccinate—TT vaccine, there was a significant reduction in total infusions of either morphine or heroin, respectively, when compared to vaccinated controls.

In addition, Mendez et al. [47] examined the effects of morphine-6-hemisuccinate—TT vaccine on opioid and non-opioid drug-induced antinociception in mice. The found that antibodies produced by the morphine-6-hemisuccinate—TT vaccine did not alter the antinociception induced by different doses of morphine. In this study, mice immunized with the morphine-6-hemisuccinate—TT vaccine displayed significant attenuation in the thermal antinociception (tail-flick test) induced by 0.5, 1, or 5 mg/kg morphine. These results are in accordance with previous research reporting that different models of vaccines can attenuate the antinociceptive effect of up to 2 mg/kg morphine or heroin, in the tail-flick and the hotplate test. Conversely, the morphine-6-hemisuccinate—TT vaccine was not able to decrease the antinociceptive effect induced by higher doses of morphine (10 and 20 mg/kg). Moreover, these antibodies did not affect the ability of non-opioid drugs (different doses of gabapentin, tramadol, or fentanyl) to block thermal pain antinociception. Finally, the combination of morphine-6-hemisuccinate—TT vaccine and naloxone prolonged the time-course of morphine antagonism relative to either vaccination or naloxone alone.

The antibodies produced by the morphine-6-hemisuccinate—TT vaccine were unable to recognize molecules that were structurally different from morphine, such as gabapentin, and synthetic molecules that had some structural similarity to morphine, such as tramadol and fentanyl.

Next, Mendez et al. [56] developed two formulations of opioid vaccines based on the morphine-6-hemisuccinate—TT vaccine and 3-carboxymethylmorphine—TT vaccine in a hope to generate a robust immune response capable of recognizing heroin and morphine. In this regard, Balb/c mice were immunized simultaneously with the morphine-6-hemisuccinate—TT vaccine and with the 3-carboxymethylmorphine—TT vaccine. A solid-phase antibody-capture ELISA was used for monitoring antibody titre responses after each booster dose in vaccinated animals. Immunization with this vaccine combination has elicited a robust immune response with an antibody titre of 1:590,000 able to recognize heroin and morphine. These antibodies are capable of reducing the antinociceptive effects induced by doses of up to 40 mg/kg of morphine or 10 mg/kg of heroin in tail-flick and hot plate assays. This study highlighted that the combination of two vaccine formulations that generate antibodies with different but complementary characteristics would be a new therapeutic strategy aimed at reducing drug relapses.

It is worth noting that Li et al. [50] have designed and synthesized a novel structural formulation of a morphine–KLH vaccine that generated a robust and sustained humoral immunological response to morphine. The hapten was prepared by reacting morphine base with glutaric anhydride in the presence of a 4-dimethyl-aminopyridine catalyst. The 3,6-diester was selectively hydrolysed with sodium hydroxide to obtain morphine-6-hemiglutarate. The latter was coupled to BSA or KLH using carbodiimide as a coupling reagent. In fact, the morphine-6-hemiglutarate contains one more carbon atom than morphine-6-hemisuccinate, and the linker part of the conjugate is longer. Antibody titres in plasma were measured using an ELISA. A competitive ELISA was used to assess the selectivity of the antibodies. The 6-glutarylmorphine—KLH conjugate generated a high antibody titre response. This vaccine displayed specificity for both morphine and heroin, but the anti-morphine antibodies could not recognize dissimilar therapeutic opioid compounds, such as buprenorphine, methadone, naloxone, naltrexone, codeine, and nalorphine. The morphine antibody significantly decreased morphine-induced locomotor activity in rats after immunization. Importantly, rats immunized with this vaccine did not exhibit a heroin-primed reinstatement of heroin seeking when antibody levels were sufficiently high. These results suggest that immunization with a novel vaccine is an effective means of inducing a morphine-specific antibody response that can attenuate the behavioural and pharmacological effects of heroin.

## 4. Immunotherapy against Heroin, Oxycodone, and Fentanyl

### 4.1. Immunotherapy against Heroin

Illicit opioid use has a major impact on society [57,58,59,60,61,62]. It involves highly increased mortality and morbidity, marginalization, and criminal behaviour. Heroin addiction involves long-standing changes in the mesolimbic dopaminergic system including tolerance for the euphoric effects and sensitisation for the incentive to take drugs. It is well documented that the neural mechanisms of drug addiction display a persistent vulnerability to drug-relapse in addicts after prolonged drug-free periods as a result of repeated drug use.

Heroin is rapidly metabolized to 6-monoacetylmorphine and then to morphine in serum by cholinesterases. Both of these compounds are MOR agonists. While diacetylmorphine and 6-monoacetyl-morphine readily cross the blood–brain barrier, morphine itself is much slower to do so. Thus, heroin could be considered a prodrug, which facilitates the entry of morphine into the brain. The metabolic pathway markedly influences the development of anti-heroin hapten antibodies. Attempts to generate such antibodies for immunotherapy should target not only heroin but its active metabolites as well. Thus, a successful heroin vaccine involves the elicitation of antibodies capable of kinetically sequestering heroin and 6-*O*-acetylmorphine in the peripheral circulation, thereby reducing these compounds from entering the brain.

In this part we summarize the efforts of two research teams, Dr. Kim Janda and co-workers at The Scripps Research Institute in La Jolla, Ca. and the research group of Dr. K. C. Rice and Dr. Gary Matyas at the NIH.

The research team of Dr. Kim D. Janda selected a novel approach in the hapten design: the linker was coupled to the nitrogen of the piperidine ring of heroin [63,64,65]. They utilized an amide bond for the attachment of the hapten to the carrier protein instead of an ester, and they used a modular linker to allow for facile comparison between different haptens.

The reductive amination of *N*-Boc-4-aminobutyraldehyde (Boc-NH-(CH_2_)_3_-CHO) with 3,6-di-*O*-acetylnormorphine (**24**) using NaBH(OAc)_3_ gave Boc-protected amine (**25**) in a 65% yield from heroin. The acidic deprotection of the yielded primary amine (**26**) was coupled with the *N*-hydroxy-succinic ester of *S*-tritylmercapto propionic acid to give trityl protected heroin hapten (**27**) in a 70% yield from the Boc-protected amine. From this protected amine, the analogous morphine hapten was also prepared. The trityl protecting group of heroin/morphine haptens was then removed under acidic conditions (trifluoroacetic acid) to give thiols (**28ab**) followed by preparative HPLC purification and conjugation to maleimide-activated KLH or BSA to give immunoconjugates (**29ab**).

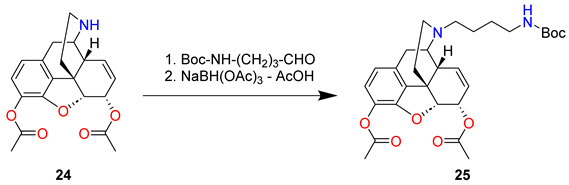



The antibody specificity for heroin, 6-*O*-acetylmorphine, and morphine was determined by competition ELISA. Antisera from rats vaccinated with heroin hapten immunoconjugate (**29a**) bound 6-*O*-acetylmorphine with high affinity, while heroin and morphine were bound with decreased affinity. On the other hand, antisera from rats vaccinated with morphine hapten immunoconjugate (**29b**) bound only morphine and heroin with high and low affinity, respectively. Vaccination selectively blocks the thermal and mechanical antinociceptive effects of heroin in rats. Systemic injection of heroin (1 mg/kg, sc) resulted in decreases in both thermal nociceptive sensitivity, as measured by a hot plate test, and mechanical sensitivity as measured by von Frey filament testing. This was fully reversed in the heroin hapten vaccine (**29a**) group. The morphine hapten vaccine (**29b**) significantly blunted the thermal nociceptive effects of heroin. The ability of each vaccine to hinder the acquisition of heroin self-administration in rats was investigated. Rats vaccinated with morphine hapten immunoconjugate (**29b**) showed similar ability to controls to acquire heroin self-administration. However, the majority of heroin hapten immunoconjugate (**29a**) vaccinated rats failed to maintain pressing for heroin. 
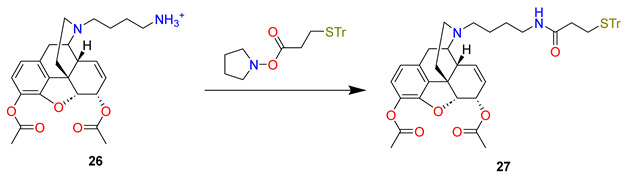


In summary, the heroin–KLH hapten–protein immunoconjugate, in formulation with the T-helper 2 (Th2) adjuvant alum (insoluble aluminium salts), generated high antibody titres specific for heroin and its deacetylated metabolites 6-*O*-acetylmorphine and morphine. Furthermore, the blockade of the heroin antinociceptive effect and self-administration studies in vaccinated rats corroborated the in vivo efficacy of this heroin vaccine. Janda et al. speculated that the success of this vaccine is attributed to its dynamic nature: the hapten is able to mimic heroin metabolism, presenting epitopes similar to heroin, 6-*O*-acetylmorphine, and morphine. In contrast, the deacetylated, morphine-like hapten conjugate elicited antibodies with poor affinity for heroin and 6-*O*-acetylmorphine. 
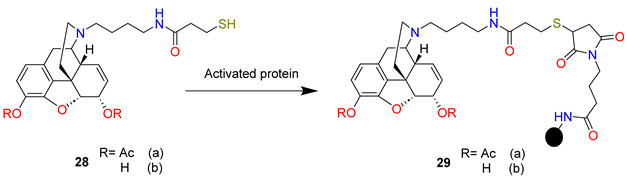


In addition, Janda et al. [66] reported the preparation of a more hydrolytically stable variation on their previous heroin hapten in which the labile C-3-acetate is replaced with a C-3-acetamide isostere **34**. The 3-acetamide would resist hydrolysis, thus preventing the usual conversion to the deacetylated hapten. Expectedly, the antibody affinity profile for heroin and its metabolites may be altered markedly and improved hapten stability may elevate anti-heroin antibody titres. The conversion of one or both of the labile esters in the heroin hapten to amides would significantly increase vaccine half-life, potentially permitting long-term storage.

A novel synthesis of a 3-aminomorphine precursor has been elaborated. Morphine base was converted to C-3 trifluoromethanesulfonate by the selective triflation of the phenolic hydroxyl group with *N*-phenyltriflamide. The C-6 secondary alcohol was protected with a *tert*-butyldimethylsilyl group (**30**). The key step of the synthesis involved a palladium-catalysed Buchwald amination of aryl triflate with benzophenone imine resulting in the replacement of the triflate to afford the imine intermediate (**31**). The hydrolysis of the imine intermediate with hydroxylamine-HCl yielded the silyl ether of 3-amino-3-desoxymorphine. The latter converted to diacetylated compound (**32**) with acetyl bromide in concomitant desilylation and diacetylation reactions. After *N*-demethylation and *N*-alkylation, the hapten was conjugated with KLH via sulfhydryl-maleimide coupling (**33** → **34**). 
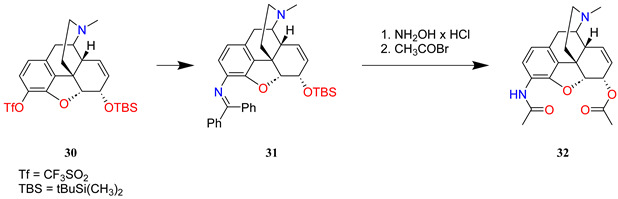


To compare the performance of the new hapten and adjuvant with the heroin hapten KLH vaccine, mice were immunized with either hapten–protein conjugate **29a** or **34** in formulation with alum or alum + CpG ODN 1826. Vaccine efficacy was measured by ELISA to assess anti-heroin antibody titres and competitive ELISA to assess antibody affinity and specificity for heroin and its metabolites. Although both haptens appear equally immunogenic, 6-*O*-acetylmorphine affinity decreases significantly (∼1000-fold) when the C-3-acetamide is incorporated into the hapten. Probably the acetamide cannot be hydrolysed, precluding conversion to a 6-*O*-acetylmorphine, or morphine-like epitope, which both contain the C-3 phenolic hydroxyl groups. In contrast, 12-fold lower heroin affinities in the **34** vaccine cannot be explained in the same manner because heroin is the parent compound in the metabolism pathway. The lower heroin affinities are likely a result of the 3-acetamide hapten presenting a slightly altered epitope from the native heroin hapten (3-acetate) in **29a**. Although the C-3-acetamide vaccine displayed enhanced resistance to hydrolysis, its significantly reduced antibody affinity for 6-*O*-acetylmorphine limits its therapeutic potential as a heroin vaccine. 
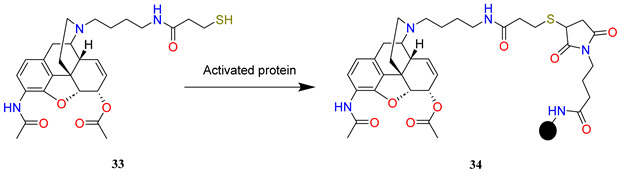


In a comprehensive study, Janda et al. [67] have reported on anti-heroin vaccine optimization with reference to the hapten structure, carrier proteins, and new adjuvants. 

In this regard, the reductive amination of Boc-protected 4-aminobutanal with 3,6-diacetylnormorphine (**24**) followed by TFA deprotection afforded the key intermediate *N*-epsilon-aminobutyl-diacetylnormorphine (**26**). Acylation with β-tritymercaptopropionic acid *N*-hydroxy-succinimide ester and deprotection yielded the first-generation thiol hapten (**27**). Novel second generation carboxylic acid haptens were prepared performing the acylations with succinic acid *tert*-butyl ester. After deprotection, two carboxylic acid haptens were obtained, the heroin-COOH hapten (**35a** HerCOOH) and 6–*O*-acetylmorphine-COOH hapten (**35b** 6AMCOOH). The third hapten was prepared in the coupling reaction of the heroin-COOH hapten (HerCOOH) and β-alanine dipeptide (**36** HerdBA). Thiol and carboxylic acid haptens were coupled to surface lysines of carrier proteins via thiol-maleimide or amide couplings, respectively. Three of the most commonly used carrier proteins, KLH, diphtheria toxoid (DT), and TT, were investigated in the context of the heroin conjugate vaccine. 
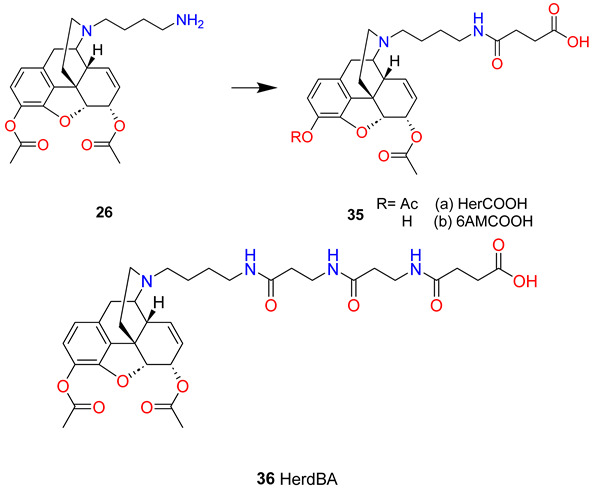


In comparing the proteins as HerSH conjugates, TT displayed the best performance in attenuating heroin-induced antinociception in mice and produced the greatest anti-hapten titres. Heroin hapten design was greatly improved through the conversion of the terminal thiol (HerSH) to a carboxylic acid (HerCOOH). This modification enabled more efficient and reliable protein coupling, producing a more efficacious immunoconjugate with a higher epitope density. Bioconjugation methods and linker structures were also compared through the testing of TT conjugates of the HerSH (**27**), HerCOOH (**35a**) and HerdBA (**36**), and HerCOOH hapten demonstrated the greatest efficacy. Furthermore, the HerCOOH hapten contains a shorter linker with less chemical functionalities relative to HerSH and HerdBA. Following antigen processing, the HerCOOH hapten linker likely interferes minimally with the immune presentation of a heroin-like epitope. The β-alanine dipeptide linker was not effective, which may be explained by the fact that beta-alanine is not naturally found in proteins, thus hampering immune processing.

Regarding the antinociceptive property, the results obtained in the mouse hot plate and tail-immersion assays indicated that the 6-*O*-acetylmorphine and the heroin haptens were comparable in efficacy. Furthermore, ELISA results corroborated this finding because sera from both groups bound both 6-*O*-acetylmorphine and heroin haptens to a similar degree; however, the 6-*O*-acetylmorphine conjugate elicited antibodies with a slightly reduced capacity to bind the heroin hapten. From an efficacy standpoint, both 6AMCOOH- and HerCOOH-TT conjugates behaved similarly, implying that the heroin hapten likely hydrolyses in vivo almost completely to the 6-*O*-acetylmorphine hapten. The immunization of mice with an optimized heroin–TT conjugate formulated with adjuvants alum and CpG oligodeoxynucleotide (ODN) generated heroin “immunoantagonism”, reducing heroin potency by >15-fold. Moreover, the vaccine effects proved to be durable, persisting for over eight months. Finally, the lead vaccine was effective in *Rhesus* monkeys, generating significant and sustained antidrug IgG titres in each subject. Based on these results an efficacious heroin vaccine has been identified through the optimization of the adjuvant (CpG ODN+ alum), carrier protein (TT), and hapten (HerCOOH).

Janda et al. [68,69,70] have also synthesized methanesulfonate esters of heroin haptens; a C-3 methanesulfonate 6-*O*-acetate (**37**) and a 3,6-dimethanesulfonate (**38**) as carboxyl-isosteres of heroin then compared them to the standard heroin hapten (**28** HAc) through vaccination studies.

The authors hypothesized that the sulfonate group would alter the immune recognition of the heroin–hapten conjugates because of its polar character but this change does not disrupt the immune recognition of heroin and 6-*O*-acetylmorphine. The mono-sulfonate hapten (**37** HMsuAc) was prepared from the second generation heroin hapten and it contains a C-6 *O*-acetyl group. The reaction of this hapten with methanesulfonic acid chloride in the presence of triethylamine yielded the 6-*O*-acetyl-C-3 methanesulfonate ester. The 3,6-disulfonate hapten (**38** H3,6Ds) was prepared with a shorter nitrogen substituent. Normorphine was *N*-alkylated with β-bromopropionic acid *tert*-butyl ester and this compound was converted to the 3,6-dimethanesulfonate with methanesulfonic acid chloride in the presence of triethylamine. Cleaving the ester protecting group, the free carboxylic acids were coupled to the carrier protein CRM197 and the resulting CRM-immunoconjugates were used to vaccinate Swiss Webster mice. The HAc vaccine group presented with greater titres compared with the HMsuAc and H3,6Ds groups. Binding studies demonstrated that the highest affinity anti-heroin antibodies were generated by the HMsuAc vaccine followed by the HAc and H3,6Ds vaccines, respectively (HMsuAc > Hac ≫ H3,6Ds). However, neither the HMsuAc nor H3,6Ds vaccines were able to generate high affinity antibodies to the active metabolite 6-acetylmorphine, in comparison to the HAc vaccine. Blood–brain bio-distribution studies supported these binding results with vaccine efficiency following the trend HAc > HMsuAc ≫ H3,6Ds. 
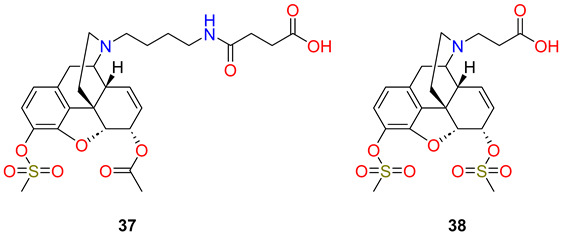


In a study from the same research group, Belz et al. [71] reported an interesting observation about the improvement of heroin vaccine’s efficacy through hapten deuteration. Diacetylnormorphine was converted in two steps to 3,6-diacetyl-*N*-carboxyethylnormorphine by *N*-alkylation with β-bromopropionic acid *tert*-butyl ester, and then this ester was cleaved with trifluoroacetic acid. The hapten (**39a** R=CH_3_) was coupled to the carrier protein KLH. These reactions were performed with hexadeuteroacetyl-normorphine starting material to obtain the deuterated hapten (**39b** R=CD_3_).

The authors did not measure real differences in affinity to 6-*O*-acetylmorphine between the two vaccines, but large differences in affinity to heroin were found. The deuterated hapten vaccine generated tighter binding affinity to heroin, whereas with the diacetyl hapten vaccine poor affinity was measured to heroin. The hot plate and tail-flick assays were carried out to compare the antinociceptive efficacy of the two vaccines: deuterated hapten vaccine outperformed the diacetyl hapten vaccine in both hot plate and tail-flick assays. The improved antinociception results for the deuterated hapten vaccine are readily explained by the improved titres. 
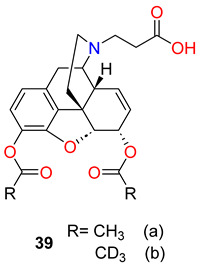


Liposomal formulation strategies have also been applied. In this regard, Matyas et al. [72] investigated the utility of producing liposomal formulations containing monophosphoryl lipid A [L(MPLA)] that could induce high levels of specific antibodies to opioid haptens that might be useful as a candidate vaccine to heroin and similar opioids.

The research team selected four haptens, MorHap (**40**), OMAHap (**41**), HerHap (**42**), and 6-AcMorHap (**43**), which were conjugated to TT and mixed with liposomes containing MPLA. Although each of the conjugate formulations was highly immunogenic in mice, the relative respective anti-hapten titres observed differed greatly, with MorHap (6,500,000) > HerHap (3,000,000) > 6-AcMorHap (500,000) > OMAHap (100,000). In another immunization strategy, a hydrophobic 23 amino acid immunogenic peptide derived from the membrane proximal external region of gp41 from the HIV-1 envelope protein was embedded as a carrier in the outer surface of L(MPLA) to which was conjugated a 15 amino acid universal T cell epitope and a terminal heroin hapten analogue. Under the conditions used, this resulted in a mean titre of 12,800 to HerHap, but no detectable antibodies were induced to OMA-Hap. The authors have concluded that L(MPLA) serves as a potent adjuvant for inducing antibodies to synthetic heroin haptens. 
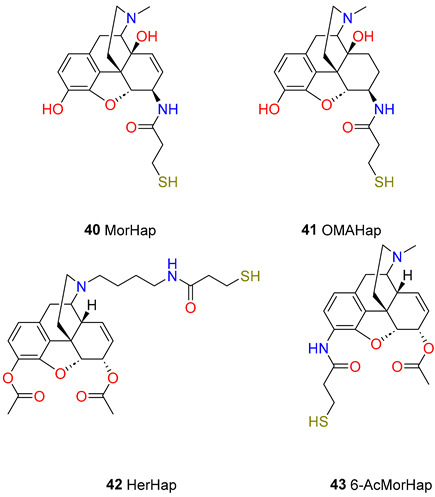


The next attempt was performed to generate new opioid haptens. In this respect, Rice research group [73] reported a synthetic sequence for the preparation of three haptens, PrOxyHap (**44**), DiPrOxyHap (**45**), and DiAmHap (**46**). PrOxyHap contains a C6α-2-oxopropyl substituent and a C-3-amino morphinan skeleton and the amino group is acylated with β-tritylmercapto propionic acid. DiPrOxyHap contains 2-oxopropyl substituents at positions of C-3 and C-6 and the 17-nitrogen is alkylated with a δ-aminobutyl group, but the primary amino group is acylated with β-tritylmercapto propionic acid. DiAmHap has 3,6α-diacetylamino substituents and the 17-N substituent is the same as in the compound DiPrOxyHap. Dihydrocodeinone, dihydromorphinone, and 3-desoxydihydromorphinone were starting materials for the hapten syntheses. 

The immunization of mice with these haptens coupled to TT (maleimide-thiol coupling) mixed with liposomes containing monophosphoryl lipid A induced high titer antibodies to each of the haptens. Immunization with DiAmHap produced both a higher titre of antibodies than DiPrOxyHap and protected mice against heroin challenge in the hot plate assay, whereas DiPrOxyHap immunization did not. Competitive ELISA was used to assess the ability of the anti-hapten antibodies to bind to heroin, 6-acetylmorphine, and morphine. Sera from mice immunized with 6-PrOxyHap were inhibited by heroin, 6-acetylmorphine, morphine, and codeine. Morphine and codeine did not compete for the binding of sera from DiPrOxyHap immunized animals to DiPrOxyHap; 6-PrOxyHap induces antibodies that bind more effectively to heroin, 6-acetylmorphine, morphine, and codeine than antibodies from DiPrOxyHap immunized mice. 
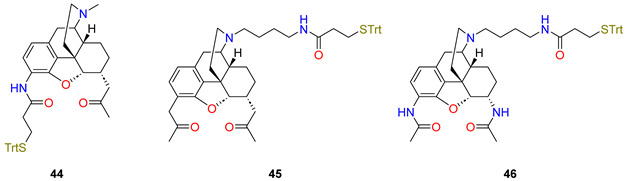


In a study by Torres et al. [74,75], the elaboration of an optimized procedure for the preparation of a BSA and a specific heroin hapten (**40** MorHap) conjugate has been carried out. In this regard, a heroin hapten was coupled to BSA using maleimide-thiol chemistry. MorHap-BSA conjugates were synthesized with highly reproducible results and in high yields. The highest antibody binding was obtained with MorHap-BSA conjugates containing 3–5 haptens. In general, the chemical structure of the hapten and the number of covalently attached haptens per molecule of the carrier protein are critical for the generation of high antibody titres.

**Scheme 2:**
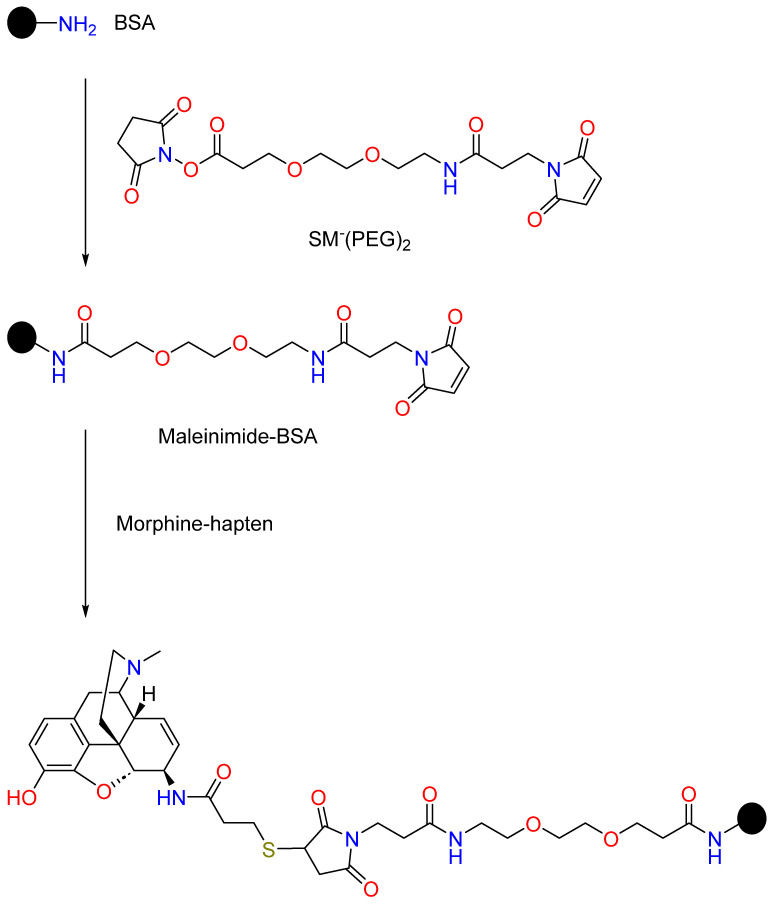
Maleimide-BSA– conjugate.

In this work, Torres et al. used MorHap, a heroin/morphine hapten, as a model hapten to optimize its attachment to a carrier protein, BSA. Earlier, MorHap (**40**) was attached to TT and used as a candidate vaccine that induced high titre antibodies, which reacted with heroin and its metabolites, 6-acetylmorphine and morphine, and protected mice from heroin challenge in an antinociception assay. The maleimide-thiol reaction was employed to prevent an oligomerization reaction. This coupling chemistry utilizes an *N*-alkylated maleimide linker with two PEG units and a *N*-hydroxysuccinimide (NHS) ester on one end for coupling to the lysines of BSA and maleimide functionality on the other end for coupling to thiols. The thiolated MorHap was conjugated to BSA in a two-step reaction using SM-(PEG)_2_. Surface lysines of BSA were first reacted with the NHS ester end of the linker to give an activated maleimide-BSA intermediate. The subsequent step used the Michael addition of MorHap to the maleimide end of the BSA intermediate. Trityl protecting group of the hapten was cleaved by trifluoroacetic acid followed the Michael addition of the free SH group to the double bond of the bovine serum albumin–maleimide conjugate (Scheme 2). The authors reported a 20–25% yield for the conjugate. 

Matyas et al. [76] hypothesized that DiAmHap (**46**), a chemically stable *N*-substituted heroin-like hapten presented to the immune system with a carrier known to be highly effective in humans (TT) and a potent adjuvant that has been widely used in humans (liposomes containing monophosphoryl lipid A), can induce high titres of cross-reacting antibodies to heroin or to its major active metabolites. The research team studied two hydrolytically stable haptens: DiAmHap, an *N*-substituted hapten, and MorHap, a C6-linked hapten which is similar to both 6-acetylmorphine and morphine. Each of these haptens induced antibodies that exhibited cross-reactivity either with heroin and each of its metabolites (DiAmHap), or with 6-acetylmorphine and morphine but not heroin (MorHap). Each immunized mouse or non-immunized control mouse was tested for antinociceptive effects of sc. injected heroin in the hot plate test. All of the mice immunized with MorHap, and with DiAmHap, had a reduced heroin effect, and the effects of each type of immunization resulted in a significant inhibition of heroin-induced antinociception.

Another study by Jalah et al. [77] demonstrated that MorHap (**40**), a heroin/morphine hapten, conjugated to TT and mixed with liposomes containing [L(MPLA)] as an adjuvant, partially blocked the antinociceptive effects of heroin in mice. MorHap is similar to the C6-linked morphine-based haptens previously described, because it contains an amide linker at the C6-position, which is hydrolytically stable in contrast to the ester functional group at the C-6 position. The immunization of mice with these conjugates mixed with L(MPLA) induced very high anti-MorHap IgG peak levels of 400–1500 μg/mL that bound to both heroin and its metabolites, 6-acetylmorphine and morphine. Mice injected with TT-1 showed a significant inhibition of heroin-induced antinociception compared to control mice in the tail-flick and hot plate tests.

Sulima et al. [78] elaborated the synthesis of 6-AmHap (**47**) which is a novel heroin hapten. This hapten has a 3-desoxy-3-amino-6α-amino-6-desoxydihydromorphine structure, and the C-3 amino group is acylated with the protected β-mercaptopropionic acid and the C-6 amino group is acetylated. The synthesis was elaborated from dihydromorphinone starting material. 6-AmHap was conjugated to TT and mixed with liposomes containing [L(MPLA)] as an adjuvant. The 6-AmHap vaccine induced high anti-**1** IgG levels that reduced heroin-induced antinociception and locomotive behavioural changes following repeated subcutaneous (sc.) and iv. heroin challenges in mice and rats. The 6-AmHap vaccine-induced antibodies bound to heroin and other abused opioids, including hydrocodone, oxycodone, hydromorphone, oxymorphone, and codeine. The cross-reactivity of the antibodies with other commonly abused prescription opioids suggests that the vaccine may have utility for these other opioids as well (Table 2). 
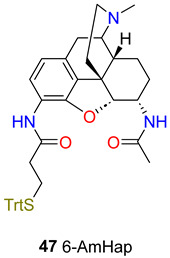

**Table 2:** Opioid haptens of heroin.

Opioid Hapten	No	Position of Hapten	Immunogen	Antibody Titres Specific	** Cross-Reactivities	Reference
Heroin hapten (HAc)	28a	N-17	28a-maleimide activated KLH	6-*O*-Ac-Mor *, heroin ^+^, Mor ^+^	6-*O*-Ac-Mor	[63,64,65]
Morphine hapten (N-thiol)	28b	N-17	28b-maleimide activated KLH	Mor *, heroin ^+^	Mor	[63,64,65]
C-3-acetamide analogue	33	N-17	33-maleimide activated KLH	6-O-Ac-Mor ++, heroin+	ND	[66]
HerdBA (β-alanine dipeptide linker)	36	N-17	36-TT	Ø	ND	[67]
C-3 methanesulfonate 6-O-acetate carboxyl-isoster of heroin (HMsuAc)	37	N-17	37-CRM	Heroin *	ND	[68,69,70]
3,6-dimethanesulfonate carboxyl-isoster heroin (H3,6Ds)	38	N-17	38-CRM	Heroin *	ND	[68,69,70]
HAc	28a	N-17	HAc-CRM	Heroin *	ND	[68,69,70]
3,6-diacetyl-N-carboxyethylnormorphine	39a	N-17	39a-KLH	Heroin	ND	[68,69,70]
Deuterated heroin hapten	39b	N-17	39b-KLH		ND	[71]
MorHap	40	C-6	40-TT+[L(MPLA)]		Mor, 6AM	[72,74,75]
OMAHap	41	C-6	41-TT+[L(MPLA)]			[72]
HerHap	42	N-17	42-TT+[L(MPLA)]			[72]
6-AcMorHap	43	C-3	43-TT+[L(MPLA)]			[72]
PrOxyHap	44	C-3	44-TT+[L(MPLA)]			[73]
DiPrOxyHap	45	N-17	45-TT+[L(MPLA)]			[73]
DiAmHap	46	N-17	46-TT+[L(MPLA)]		Heroin, Mor, 6AM	[73]
6-AmHap	47	C-3	47-TT+[L(MPLA)]		Heroin, and other abused opioids, including hydrocodone, oxycodone, hydromorphone, oxymorphone, and codeine	[78]
6,14-AmidoHap	48	C-14	48-TT+[L(MPLA)]/Al(OH)3			[79]
14-AmidoMorHap	49a	C-14	49a-TT+[L(MPLA)]/Al(OH)3			[79]
14-AmidoHerHap	49b	C-14	49b-TT+[L(MPLA)]/Al(OH)3		Heroin, 6AM	[79]
1-AmidoMorHap	50	C-1	50-TT+Al(OH)3-ALF43	Ø		[80]
1-AmidoMorHap epimer	51	C-1	51-TT+Al(OH)3-ALF43	6-*O*-Ac-Mor, heroin		[80]
1-Amido-DihydroMorHap	52	C-1	52-TT+Al(OH)3-ALF43	Ø		[80]
1 Amido-DihydroMorHap epimer	53	C-1	53-TT+Al(OH)3-ALF43	6-O-Ac-Mor, heroin		[80]

Abbreviations: morphine, Mor; codeine, Cod; 6-O-acetylmorphine, 6-O-Ac-Mor; *, high binding capability; ^+^, low binding capability; ++, lower binding capability; Ø, not effective; Army Liposome Formulation, ALF43; **, cross-reactivities > 10%; ND, no data.

Gutman et al. [79] reported the synthesis of three novel opioid haptens with the linker attachment site at C14, **48** (6,14-AmidoHap), **49** R = H (14-AmidoMorHap), and **49** R = Ac (14-AmidoHerHap) as novel heroin haptens. The compounds **48** and **49** are similar but with different substituents at C-6: an acetamide, a hydroxyl moiety, and an acetate, respectively. All three haptens contain a phenolic hydroxyl group at C3. The haptens were conjugated to the TT carrier protein, adjuvanted with [L(MPLA)]/aluminium hydroxide, and were tested in mice in terms of immunogenicity and efficacy. The immunization of mice resulted in antibody endpoint titres of >105 against all of the haptens. Neither of the conjugates of **48**, **49** R = H, and **49** R = Ac had induced antibodies with selectivity broad enough to recognize and bind heroin, 6-*O*-acetylmorphine, and morphine, resulting in little to no protection against the antinociceptive effects of heroin in vivo. Only the mice immunized with conjugate **49** R = Ac were partially protected against heroin-induced antinociception. The vaccine efficacy was evaluated by heroin challenge experiments in mice using the tail-immersion test; animals were challenged subcutaneously with heroin. The authors concluded that by changing the substituent at the C-6 position, the selectivity of the induced antibodies was effectively altered. Specifically, TT-48 and TT-49 R = Ac, where C-6 has been substituted by an acetate group and an amide group, respectively, induced antibodies that effectively sequestered 6-AM. Linker attachment at C14 did not induce antibodies with broad enough selectivity to simultaneously recognize and sequester heroin, 6-*O*-acetylmorphine, and morphine. 
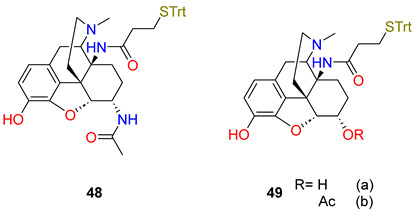


Finally, in relation to immunotherapy against heroin, Sulima et al. [80] reported the design and synthesis of the following haptens: 1-AmidoMorHap (**50**), 1-AmidoMorHap epimer (**51**), 1 Amido-DihydroMorHap (**52**), and 1 Amido-DihydroMorHap epimer (**53**). The haptens were separately conjugated to TT carrier protein using the previously optimized method. Vaccines were adsorbed to aluminium hydroxide and mixed with Army Liposome Formulation (ALF43) adsorbed to aluminium hydroxide as an adjuvant. Immunogenicity was assessed by immunizing mice and collecting sera at weeks 0, 3, and 6. Antibodies to the immunizing haptens were measured using an ELISA that used BSA–hapten conjugates as coating agents. The results showed that all of the haptens induced high antibody endpoint titres (>105 after the second vaccine dose) against their respective antigens. This suggests that all of the vaccine candidates tested are immunogenic. The in vivo efficacy of each vaccine candidate was assessed by the analgesic hot plate assay. It was found that among the conjugates tested, only TT-**51** and TT-**53** gave low % maximal possible effect (MPE) values, suggesting that mice were protected from the antinociceptive effects of heroin. The other conjugates, TT-**50** and TT-**52**, carrying epimeric haptens displayed no significant difference with the unvaccinated controls. Only TT-**51** and TT-**53** yielded antibodies that bound heroin and 6-acetyl morphine. None of the TT–hapten conjugates induced antibodies that cross-reacted with morphine, methadone, naloxone, or naltrexone. The antibodies induced by TT-**51** and TT-**53** were notably weak. This may be because neither of these haptens exactly mimicked the three-dimensional orientation of the target drugs. 
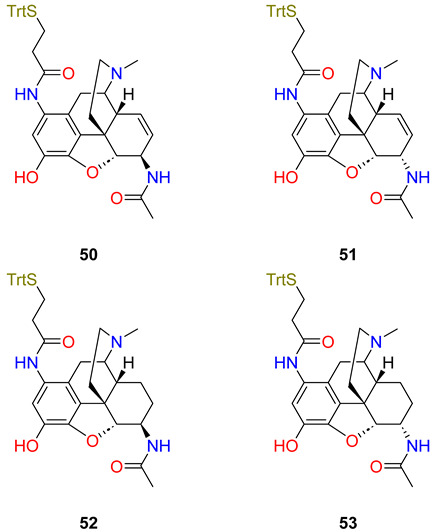


### 4.2. Immunotherapy against Oxycodone

In the past decade, the abuse of prescription opioids has increased dramatically, mainly in the United States. The rise in prescription opioids has been accompanied by an increase in the incidence of emergency department visits and opioid overdoses. Oxycodone (OxyContin) is the most commonly abused prescription opioid. Dr. M. Pravetoni and co-workers at the University of Minnesota developed vaccines against oxycodone abuse.

In this work, Pravetoni et al. [3] have synthesised and evaluated the immunologic and pharmacokinetic effects of oxycodone conjugate vaccines in rats. Vaccines based on morphine conjugates (morphine-6-hemisuccinate hapten conjugates) produce specific antibodies which bind heroin and its active metabolites 6-*O*-acetylmorphine and morphine, but they possess low affinity for oxycodone. In order to prepare an oxycodone hapten conjugate, two linkers were chosen for evaluation: hemisuccinate, because it has been used successfully with heroin conjugate vaccines, and the tetrapeptide (Gly)_4_, because preliminary studies were successful. Oxycodone was converted to the *O*-carboxymethyloxime with *O*-carboxymethyloxime x HCl salt. This oxime was coupled to tetraglycine *tert*-butyl ester (Gly_4_tBu), using a *N*,*N*′-dicyclohexyl-carbodiimide/hydroxy-benzotriazole procedure followed by acid hydrolysis to afford the oxycodone hapten (**53**). The other oxycodone hapten (**54**) was prepared in the reaction of 6-desoxy-6α-amino-14-hydroxy-dihydrocodeine with succinic anhydride using pyridine solvent. Both haptens were first conjugated to BSA to optimize reaction efficiency, then both haptens were coupled to KLH (Table 3). The oxycodone-(Gly)_4_-BSA and oxycodone-hemisuccinate-BSA conjugates had nearly identical haptenation ratios, yet the conjugate containing the (Gly)_4_ linker elicited substantially higher titres regardless of the immunogen dose. The oxycodone-(Gly)_4_-KLH immunogen elicited high titres of oxycodone-specific antibodies that were highly selective for oxycodone and its active metabolite oxymorphone and had substantially lower affinities for morphine, dihydrocodeinone, and dihydromorphinone. The vaccination of rats with this immunogen increased oxycodone binding and retention in serum. The effects of oxycodone-(Gly)_4_-KLH on oxycodone-induced antinociception in the hot plate test were studied to measure the ability of this vaccine to block a CNS-mediated opioid effect. Vaccination substantially reduced oxycodone-induced antinociception in a test of thermal nociception, highlighting its ability to attenuate a centrally mediated behavioural effect of oxycodone. Antibodies generated by oxycodone-(Gly)_4_-KLH cross-reacted with oxymorphone, an active but minor metabolite of oxycodone in rats and humans. Antibodies from the oxycodone-(Gly)_4_-KLH vaccine had no measurable affinity for methadone or buprenorphine and had IC_50_ values substantially higher for naltrexone and naloxone than for oxycodone. 
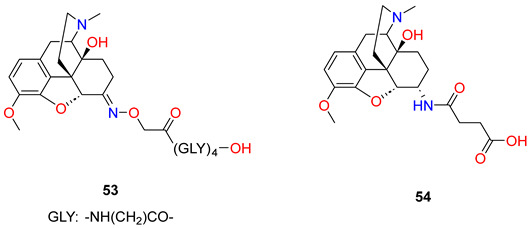

**Table 3:** Opioid haptens of oxycodone.

Opioid Hapten	No	Position of Hapten	Immunogen	Antibody Titres Specific	Cross-Reactivities	Reference
Oxycodone-(Gly)4	53	C-6	53-BSA	↑ titres		[3]
Oxycodone-(Gly)4	53	C-6	53-KLH	↑ titres Oxycod	Oxymor	[81]
Oxycodone-(Gly)4	53	C-6	53-OVA		Oxycod, Oxymor	[3]
Oxycodone-hemisuccinate	54	C-6	54-BSA	Low titre	NA	[3]
Morphine-6-O-glycolic acid-(Gly)4	55	C-6	55-KLH		Oxygly4KLH (18%)	[81]

Abbreviations: Oxycodone, Oxycod; oxymorphone, Oxymor.

Furthermore, Pravetoni et al. [81] elaborated a new morphine hapten (**55**) in which the C-6 hydroxyl group was *O*-alkylated with a bromoacetic acid ester, and then the free carboxylic group was coupled with a tetraglycine linker to KLH. The immunization of rats resulted in a morphine–KLH vaccine. The authors designed an experiment in which rats received an oxycodone–KLH conjugate vaccine targeting oxycodone and its active metabolite oxymorphone, or a morphine–KLH conjugate vaccine targeting heroin, 6-*O*-acetylmorphine, and morphine. The two vaccines were administered alone or in combination to determine whether their combined use would preserve their individual efficacies. Because heroin is rapidly converted in vivo to 6-*O*-acetylmorphine, which is considered largely responsible for its acute effects, and resulting heroin levels are quite low, 6-*O*-acetylmorphine was used as a model opioid in this study rather than heroin.

The phenolic hydroxyl group of morphine was protected with a Boc group and the C-6 hydroxyl group was *O*-alkylated with a bromoacetic acid *tert*-butyl ester using sodium hydride for deprotonation. Cleaving the protecting groups, morphine-6-*O*-glycolic acid was isolated. The morphine-6-*O*-glycolic acid was coupled to tetraglycine *tert*-butyl esters (Gly_4_tBu) using dicyclohexylcarbodiimide (DCC)/hydroxybenzotriazole (HOBt) followed by acid hydrolysis to complete the synthesis of the morphine-6-*O*-glycolic acid-(Gly)_4_—conjugate (**55**). Haptens for use in the vaccines were conjugated to KLH because this protein is highly immunogenic and acceptable for use in humans. Rats were immunized and were administered i.p. in CFA. Immunization with morphine–KLH alone elicited serum antibodies that were highly specific for heroin, 6-*O*-acetylmorphine, and morphine. Immunization with oxycodone–KLH alone elicited serum antibodies that were highly specific for oxycodone and oxymorphone. Competition ELISA values showed high relative affinities for oxycodone and oxymorphone, low relative affinity for heroin, 6-*O*-acetylmorphine, or morphine, and negligible cross-reactivity with the (Gly)_4_ linker alone. Rats immunized with the combination of morphine–KLH and oxycodone–KLH developed serum antibody titres against each immunogen that were higher than in rats vaccinated with monovalent vaccines alone. These titres were greater than could be accounted for simply by adding the increase in titres attributable to the cross-reactivity of the antibodies as determined by ELISA. 
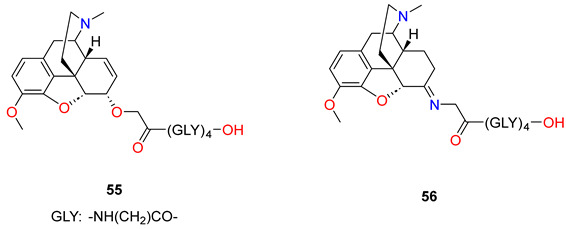


Next, Pravetoni et al. [82] prepared oxycodone-based and dihydrocodeinone-based haptens which were conjugated to KLH to generate immunogens that would recognize both oxycodone and dihydrocodeinone. In prior studies, Gly_4_ was found more suitable than a shorter linker for an oxycodone hapten. For these reasons, this linker was used to generate tetraglycine-containing hydrocodone haptens. In general, the immunogenicity of hapten–protein conjugate vaccines is improved with a higher ratio of haptenation, so the authors investigated if thiol-based maleimide conjugation would improve haptenation ratios compared to the carbodiimide method of conjugation. The oxycodone carboxymethyloxime hapten was coupled to linker *S*-tritylcysteamine using the dicyclohexylcarbodiimide/hydroxybenzotriazole (DCC/HOBt) procedure. The trityl protecting group was removed with acetic acid (AcOH) and trifluoroacetic acid to give the free thiol hapten (**57**) which was conjugated to maleimide-activated KLH (mKLH). To generate a dihydrocodeinone vaccine, a hapten was synthesized from dihydrocodeinone using the same reaction as in the case of oxycodone, and dihydrocodeinone carboxymethyloxime was prepared. This oxime was attached to KLH with tetraglycine linker (**56**). The immunogen oxycodone–KLH conjugate elicited higher serum antibody titres than those elicited by the dihydrocodeinone–KLH conjugate. The immunogen oxycodone–KLH conjugate generated a stronger blockage of hydrocodone analgesia than the immunogen dihydrocodeinone–KLH conjugate. The immunogenicity of the oxycodone–KLH conjugate and oxycodone–mKLH (**58**) conjugate were tested in rats. Vaccination elicited higher oxycodone-specific serum antibody titres generated by the oxycodone–KLH conjugate compared to oxycodone–mKLH conjugate. However, serum antibodies directed against the immunogen oxycodone–mKLH conjugate recognized oxycodone-Gly_4_ hapten (**53**) and serum antibodies against the immunogen oxycodone-Gly_4_-KLH-recognized hapten (**57**). These data suggest that immunogens oxycodone-Gly_4_-KLH and (**58**), despite their different linkers, generate antibodies with similar specificity.

The immunogen oxycodone-Gly_4_-KLH elicited serum antibody titres which were significantly higher than those elicited by the dihydrocodeinone-Gly_4_-KLH conjugate. The immunogen oxycodone-Gly_4_-KLH generated a stronger blockade of hydrocodone analgesia than the immunogen dihydrocodeinone-Gly_4_-KLH. Vaccination with oxycodone-Gly_4_-KLH comparably attenuated both oxycodone and hydrocodone antinociception in rats, but fentanyl analgesia was preserved. 
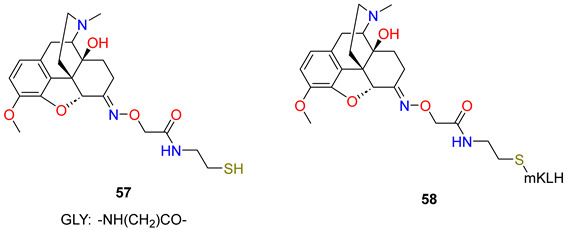


### 4.3. Immunotherapy against Fentanyl

The introduction of fentanyl and its analogues to the illicit drug market can be traced back to the seventies and eighties, when they appeared in illegal drug products that resulted in accidental overdoses [83,84]. While fentanyl is currently the most popular illicitly manufactured opioid, there are numerous derivatives that can be produced to evade distribution crimes. As mentioned in the introduction, the efficacy and low-cost synthetic procedures of fentanyl have enabled the creation of dangerous “designer drug” analogues of comparable or even higher potency than the parent drug, yet they escape toxicology screening. Indeed, the synthetic opioid fentanyl initially was used for the adulteration of heroin, but recently fentanyl and its illegally manufactured fentanyl analogues completely substituted heroin in the illicit drug trade [85]. Pharmacologically, these compounds display a fast onset of action, much more potent than heroin, and a fast euphoric effect as well as pain relief action. However, they can cause life-threatening respiratory depression within two minutes when administered intravenously. It is worth noting that managing the toxicity of such compounds is required to forgo the therapeutic dose of naloxone, namely, a five-times naloxone dose is needed to overcome the toxic effects [85]. Owing to these characteristics, fentanyl and its analogues have contributed to the growing number of overdose deaths, which is reflected in recent data reporting the number of fatalities linked to an overdose of these opioids. In this section, we unravel an effective immunotherapy for reducing fentanyl-induced OUD drugs, though these treatment tools were evaluated at the preclinical level. In the hope of putting an end to the current opioid overdose deaths created by illicitly manufactured fentanyl, in the last decade, vaccines against fentanyl were developed first by the Janda group [86].

The hapten against fentanyl was prepared from 4-phenylamino-*N*-beta-phenylethyl piperidine which is a deacylated fentanyl molecule. The reaction of this secondary amine with glutaric acid anhydride yielded a carboxylic acid and the *N*-hydroxysuccinimide ester was coupled to TT and BSA in the presence of EDC. The TT conjugate was combined with the adjuvants alum and CpG oligodeoxynucleotid 1826. Mice were immunized with the TT–fentanyl hapten conjugate; it induced very high anti-fentanyl antibody midpoint titres by ELISA of >100,000. In order to evaluate the effectiveness of the developed vaccine, the mice hot plate and tail-immersion antinociceptive assays were used. The vaccinated animals, compared to the control, have shown much less sensitivity to the antinociceptive effect of fentanyl, as indicated by a 30-fold shift to the right in the fentanyl dose–response curve. In addition, lethal doses of fentanyl were safely administered to vaccinated mice, but unvaccinated mice experienced a fatality rate of more than 50% [86]. It is noteworthy that the fentanyl vaccine displayed significant cross-reactivity with fentanyl derivatives such as 3-methylfentanyl and α-methylfentanyl. Immunized mice showed protection from these derivatives in hot plate and tail-immersion tests. 
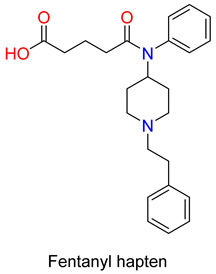


The same group has also developed and tested an admixture vaccine and its ability to protect against both drugs [87,88]. Heroin and fentanyl haptens were conjugated to the KLH protein. Antibody titres detected in the admixture were approximately half the level detected in the individual Her–KLH vaccine, as predicted based on the amount of Her–KLH immunoconjugate employed in each group, but antibody affinity remained at similar levels. Based on potency ratios, both the admixture and the individual vaccines increased the heroin ED_50_ (effective dose to produce 50% effect) values by 2–6 times for both drugs and antinociceptive tests compared to controls. In addition, they have reported on an improved admixture immunization strategy that employs carrier protein and TT, and then combines two individual immunoconjugate vaccines to target heroin and fentanyl. In this study, the admixture vaccine appeared to sequester both drugs and proved efficient in antinociceptive animal tests in comparison to individual drug vaccines [87]. Furthermore, the synthesis of a dual hapten, which contains a heroin and fentanyl moiety chemically connected to produce a single chemical epitope for antibody production to both drugs was reported [89,90]. To enhance the efficacy of the dual hapten vaccine, the research team utilized a genetically modified cross-reacting material (CRM197), rather than KLH as a carrier protein. The hapten design and carrier protein linker were optimized to produce a more stable and efficacious epitope for antibody production. Fentanyl hapten was coupled to the piperidine nitrogen of 3-amino-6-acetamido-morphinan skeleton. In the dual hapten, the carrier protein linker was connected to the morphinan C-3 amine position. The heroin hapten contains hydrolytically stable amide functional groups at the C-3 and C-6 positions. The free amine group at the C-3 position was acylated with 1,5-pentanedicarboxylic acid *tert*-butyl ester, and removing the protective group the free carboxylic acid was conjugated with immunogenic carrier protein CRM197. Mice were injected with the heroin CRM vaccine, fentanyl CRM vaccine, and dual hapten-CRM vaccine, and the antinociceptive activity of the antibodies was determined in hot plate and in tail-flick tests [89]. The heroin vaccine produced a significant effect of a three-fold shift in both the hot plate and tail-flick tests, while the fentanyl vaccine displayed a more varied response between the two tests. The dual vaccine produced a 1.5-fold and 3-fold shift when exposed to heroin and fentanyl. 

In a study carried out on non-rodent species, namely cynomolgus monkeys, five groups of cynomolgus monkeys were immunized with a formulation containing a combination of heroin- and fentanyl-hapten immunoconjugates formulated with both inulin and alum adjuvants [91]. Over the course of the 14-week immunization period, serum antibodies against heroin or fentanyl were monitored by ELISA. At weeks 14 and 22, where titre levels were at a maximum, the CpG55.2 + alum showed the highest titre levels against heroin, while at week 14, modified δ-inulin showed the highest titre levels against fentanyl [92]. 
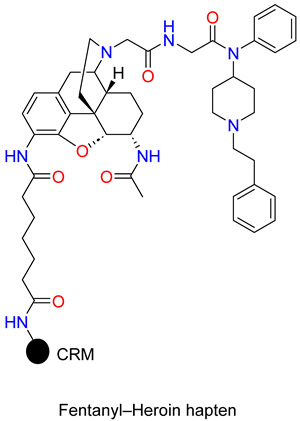


Another medicinal chemistry strategy for developing a vaccine against fentanyl was obtained by the synthesis of the fentanyl hapten through modification carried out on the piperidine nitrogen. In this regard, the beta-phenylethyl substituent was replaced with the beta-aminoethyl group [92]. The amino group was converted to a glutaric acid monoamide in the reaction with glutaric anhydride. The free carboxylic group was coupled with tetraglycine tert.butyl ester using a benztriazole (HBTU) coupling reagent and Hunig’s base. *Tert*-butyl ester was cleaved by acid hydrolysis. The hapten was conjugated to either the native decamer KLH carrier protein or the GMP grade subunit KLH (sKLH). In rats, fentanyl-sKLH attenuated the antinociceptive effect of fentanyl in the hot plate assay as well as fentanyl-induced respiratory depression or bradycardia over a range of cumulative sc. fentanyl doses. Fentanyl–KLH selectively reduced the antinociceptive effects of fentanyl, but did not alter the heroin- or oxycodone-induced antinociceptive effects [93]. The same group has also shown that immunization with vaccines produced by novel fentanyl-based haptens conjugated to carrier proteins reduced the antinociceptive effect and the respiratory depression and bradycardia evoked either by fentanyl or sufentanil in mice and rats [93]. 
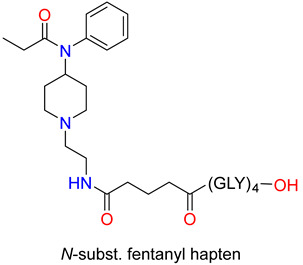


## 5. Impact of Opioid-Based Vaccination on Opioid Distribution

The distribution of heroin and its metabolite was studied after iv. administration in both non-vaccinated and vaccinated rats to determine the relative concentrations of heroin and metabolites in plasma and brain [94].

The vaccine effects on drug distribution were studied after the iv. administration of heroin. Plasma heroin concentrations were very low but were still detectable at 4 min. Heroin retention in plasma was increased 280-fold in morphine–KLH vaccinated rats compared to KLH controls at 4 min. 6-*O*-acetylmorphine and morphine concentrations in plasma were increased eight-fold in vaccinated rats compared to controls at 4 min. Vaccination with morphine–KLH did not reduce heroin distribution to the brain compared to controls, but brain heroin concentrations in both groups were low compared to 6-*O*-acetylmorphine and morphine concentrations. On the other hand, compared to controls it reduced the distribution of 6-*O*-acetylmorphine and morphine to brain in vaccinated rats by 44% and 40%, respectively, indicating that the difference was not quite significant.

After the iv. administration of 6-*O*-acetylmorphine, its retention in plasma was increased 51-fold compared to KLH controls and morphine concentrations were sixfold higher. In these experiments, rats were vaccinated i.p. with morphine–KLH in Freund adjuvant. Vaccination reduced 6-*O*-acetylmorphine concentrations in the brain by 76% compared to controls. In rats vaccinated sc. with morphine–KLH in alum, the effects were similar but smaller. Vaccination with morphine–KLH significantly reduced antinociception (hot plate test) to heroin, methadone, and oxycodone, although the effect on heroin was the greatest. These results supported the predominant role for 6-*O*-acetylmorphine in mediating heroin effects and the importance of the binding of 6-*O*-acetylmorphine by vaccine-generated antibodies in reducing those effects.

Vaccination with morphine–KLH substantially increased the retention of 6-*O*-acetylmorphine and morphine in serum. The distribution of 6-*O*-acetylmorphine to the brain was reduced by 69% compared to controls. Morphine concentrations in the brain in all groups were below assay sensitivity (<50 ng/mL). Vaccination with oxycodone-Gly_4_-KLH increased the retention of oxycodone in the serum and reduced oxycodone distribution to the brain by 66%. Oxymorphone concentrations in all groups were too low to quantitate (<5 ng/mL). Rats vaccinated with morphine–KLH and oxycodone-Gly_4_-KLH vaccines concurrently preserved the effects of the monovalent vaccines, significantly increasing 6-*O*-acetylmorphine and oxycodone retention in serum and decreasing their distribution to the brain. In general, the action of the bivalent vaccine was similar to that of the monovalent vaccine alone. The bivalent vaccine increased the serum retention of 6-*O*-acetylmorphine significantly more than the monovalent morphine–KLH vaccine. In the work by Pravetoni and co-workers, the immunization of rats with oxycodone-Gly_4_-KLH resulted in a limitation in the distribution of oxycodone to the brain while enhancing the retention of oxycodone in serum [3]. In the same animal species, the same research group has shown that immunization with oxycodone-Gly_4_-BSA enhanced the retention of oxycodone in the serum and reduced its distribution to the brain. In addition, compared to the control animal, oxycodone serum protein binding was significantly increased in rats treated with either oxycodone-Gly_4_-KLH or oxycodone-Gly_4_-BSA. In another study, the immunization of rats with oxycodone-Gly_4_-KLH induced a reduction in the distribution of either oxycodone or hydrocodone to the brain [82]. Furthermore, the distribution of oxycodone to the brain was reduced next to immunization with oxycodone-Gly_4_-KLH, but the immunization was more effective against subcutaneously administered oxycodone compared with iv. administration [95]. It is likely that the degree of distribution was influenced by the route of oxycodone administration: iv. versus sc..

With respect to fentanyl distribution next to vaccination, in both mice and rats, the fentanyl–KLH vaccination resulted in a significant increase in the plasma level of fentanyl. Compared to the control group, a 60% decrease in the antinociceptive effect of morphine was measured in the mouse hotplate assay [96]. On the other hand, in the rat hotplate assay, fentanyl–KLH vaccine administration resulted in a 93% decrease in the antinociceptive action of fentanyl compared to the control group [92]. In vaccinated rats, the brain level of fentanyl content was decreased by 30% compared with control rat brains.

## 6. Other Strategies to Generate Opioid Haptens

**Scheme 3:**
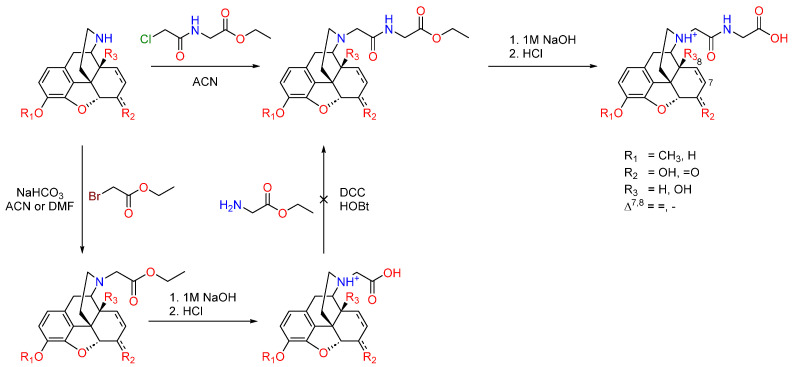
Functionalizing nor-compounds into carboxymethyl derivatives.

Köteles et al. recently prepared various *N*- and 3-*O*-opioid haptens [97,98]. To obtain the *N*-carboxymethyl nor-derivatives (normorphine, dihydronormorphine, norcodeine, dihydronorcodein, noroxymorphone, and noroxycodone) ethyl bromoacetate was used, followed by hydrolysing the esters with alkaline solution. The acidified HCl salts were further used for the coupling reactions with glycine ethyl ester in the presence of DCC and HOBt but these attempts failed. To overcome this problem *N*-acetylglycine ethyl ester was chosen as an alkylating agent and the products were hydrolysed with 1M NaOH solution. Finally, the HCl salts were isolated after acidification (Scheme 3).

**Scheme 4:**
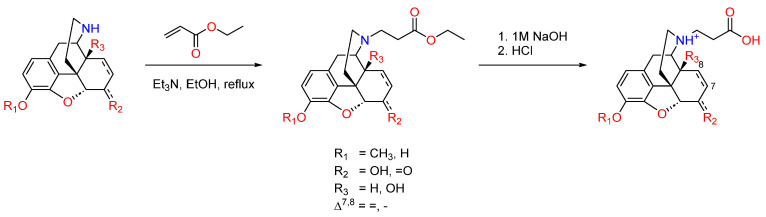
Functionalizing nor-compounds into carboxyethyl derivatives.

The carboxyethyl derivatives were obtained utilizing ethyl acrylate, isolating the products with high and excellent yields. The free carboxylic acids were prepared in a similar way to the carboxymethyl counterparts (Scheme 4).

**Scheme 5:**
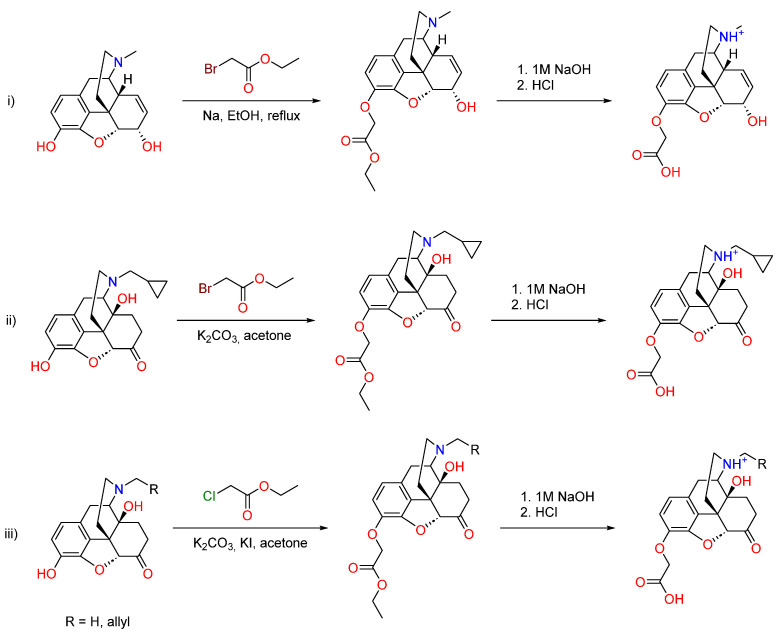
3-*O*-functionalized haptens.

Derivatizing the 3-OH containing starting compounds involved different reaction conditions: (i) metallic sodium was dissolved in absolute ethanol and ethyl bromoacetate was used to *O*-alkylate morphine HCl; (ii) ethyl bromoacetate was added to naltrexone, previously dissolved in acetone; and (iii) ethyl chloroacetate was utilized in the case of oxymorphone and naloxone, using a catalytic amount of potassium iodide. All of the esters were hydrolysed with 0.1 M NaOH solution, and during the work-up process the hydrochloride salts were isolated (Scheme 5).

As the acid–base properties of a molecule can easily influence the pharmacokinetics of a drug candidate, the protonation macro- and micro-constants were determined for the first time to study the distribution of species in the human body.

## 7. The Pharmacological Properties of Novel Opioid Haptens

The data obtained in isolated mouse vas deferens (MVD) are shown in Table 4. The efficacy indicated by the E_max_ value of the majority of tested compounds five from the eighth was below 30%, with one equal to 60, one equal to 51, and one equal to 41%. Compounds with the highest efficacy values, namely KI-184 and KI-210 were further tested in vivo. In these experiments, male Wistar rats were used in the tail-flick thermal pain model as described previously [99]. In short, a light beam is focused on the rats’ tail and the latency time (pain threshold) is measured until the animal flicks its tail away. Maximum possible effect (MPE) was calculated in percentage by the following equation: (latency-baseline)/(cut-off baseline)*100. Before the experiment, animals were familiarized with the instrument. On the day of the experiment baseline measurements were taken, then animals were treated subcutaneously and re-measured 30 and 60 min after treatment. KI-184 was tested in 10 and 20 mg/kg doses, both of which failed to produce antinociception either at 30 min (MPE% 9.9 and 3.1, respectively) or 60 min (MPE% 12.7 and 4.6, respectively). KI-210 was tested at a 20 mg/kg dose and it also failed to produce antinociceptive effect (MPE% 7.1 and 2.8 at 30 and 60 min). For each group, five animals were tested. These data indicate that even those haptens which displayed an efficacy of 40–60% in the in vitro assay failed to show an antinociceptive effect in the rat pain model. These results underscore a novel perspective on using opioid haptens of negligible analgesia in order to avoid their use for non-therapeutic purposes, namely in the illegal use or synthesis of opioids. To judge their therapeutic value in relation to the immunotherapy of OUD, future studies are needed to investigate them for immunopharmacological properties.

## 8. Conclusions

The current pharmacotherapy and psychotherapy treatments for substance use disorder (SUD), particularly OUD, are insufficient to relieve withdrawal symptoms and decrease the high relapse rates. For OUDs, opioid agonist treatment (methadone and buprenorphine) or antagonist treatment (naltrexone) is of moderate efficacy. Moreover, agonist medications are innately addictive because of their similar pharmacological profile to the opioid drug family. Recently, immunotherapy has been raised again by several research group as a promising approach for OUD, and it directly sequesters opioid drugs in the periphery instead of interacting with the targets present in CNS. The advantage of immunotherapy is that it is non-addictive because vaccines and antibodies are too large to cross the blood–brain barrier. Second-generation opioid vaccines such as the HerCOOH-TT vaccine reduce heroin antinociception at doses higher than 10 mg/kg in rhesus monkeys. Remarkably, the vaccine blocked the effects of heroin for more than 8 months with 3-month boosters. In addition, the vaccine protected against lethal heroin overdoses, which is critical in a clinical setting. Other morphine conjugate vaccines of similar design have also blocked heroin self-administration. Indeed, no heroin, oxycodone, fentanyl, or morphine vaccines have been approved for use in humans thus far. Also, to the best of our knowledge, no human trials, past or present, have been registered. As a treatment option, the development of an effective, safe and easily manufactured combination anti-heroin/HIV vaccine that could treat heroin addiction while also preventing HIV infection in those receiving the vaccine has also been proposed.

Vaccines in the treatment of OUD can be used as adjunctive therapies or in combination with other medications rather than as single modalities. Antibodies generated by the oxycodone-(Gly)_4_-KLH vaccine did not appreciably cross-react with methadone, buprenorphine, or naltrexone so that the therapeutic use of these medications as a treatment for addicts with sufficiently regular and severe opioid use would still be possible. The combination of vaccine and agonist therapy is of interest because a substantial minority of opioid addicts continue to use their opioid of choice even while on methadone maintenance therapy. A phase I/II study has been registered to investigate the effects of the oxycodone-(Gly)_4_-sKLH vaccine against oxycodone. This is a multisite study and is currently actively recruiting volunteers to assess the safety, degree of antibody production, and efficacy. Finally, the possibility of the development of opioid vaccines against specific opioid structures would guarantee better therapies to meet the needs of clinicians to treat addiction and or pain.

## Figures and Tables

**Table 1:** Opioid haptens of morphine derivative opioids (synthetic and semisynthetic phenanthrene opioids).

Opioid Hapten	No	Position of Hapten	Immunogen	Immune Specificity	Cross-Reactivities **	References
3-*O*-carboxymethylmorphine	1	C-3	1-BSA	Mor, Cod, Heroin	Cod, nor-Mor, Heroin	[1,15]
3-carboxymethylmorphine	1	C-3	1-TT	Mor, Cod, Heroin	ND	[47]
2-(*p*-aminofenilazo)-morphine	3	C-2	3-BSA	Mor, Cod, Heroin	Nor-Mor	[17]
Morphine-6-hemisuccinate	4	C-6	4-BSA	Mor, Cod, Heroin	Heroin, Mor, Cod, Nalor	[18,19]
Morphine-6-hemisuccinate	4	C-6	4-TT	Heroin, 6-*O*-Ac-Mor, M3G, M6G	Heroin, Mor	[48,49]
6-glutarylmorphine	6	C-6	6-glutarylMor– KLH	Mor, Heroin	Heroin, Mor	[50]
*N*-carboxymethyl-normorphine	8	N-17	8-BSA	Mor, NorMor, Nalor, NX	Nor-Mor, Nalor	[20,26]
2-arylazomorphine (Scheme 1 and Scheme 2)		C-2	2-arylazomorphine-BSA	Mor		[25]
2-(4-carboxyethyl-phenyl)-azomorphine	10	C-2	10-BSA		Cod, Ethylmor	[36]
2-(4-carboxy-phenyl)-azomorphine	11	C-2	11-KLH or BSA	Mor	Cod (16%), Ethylmor (11%)	[36]
Codeine-6-hemisuccinate	12a	C-6				[51]
Ethylmorphine-6-hemisuccinate	12b	C-6				[51]
Oxycodone-6-carboxymethyloxime	13	C-6	13-BSA		Oxymor	[51]
*N*-(4-aminobutyl)normorphine	17	N-17	17-BSA	Mor	Nalor	[30]
*N*-succinylnormorphine	18	N-17	18-BSA	Mor	-	[41]
*N*-substituted morphine-6-glucuronide		N-17	BTG	M6G	-	[44]
*N*-γ-aminopropyl-normorphine	21	N-17	21-BSA	Mor	Nor-Mor	[46]
*N*-γ-aminopropyl-normorphine-3-G	22	N-17	22-BSA	M3G	ND	[46]
*N*-γ-aminopropyl-normorphine-6-G	23	N-17	23-BSA	M6G	ND	[46]

Abbreviations: morphine, Mor; codeine, Cod; 6-*O*-acetylmorphine, 6-*O*-Ac-Mor; nalorphine, Nalor; naloxone, NX; **, Cross-reactivities > 10%; ND, no data.

**Table 4:** The opioid properties of novel haptens analogues in isolated mouse vas deferens.

Opioid Hapten	Chemical Structure	E_max_ (%)	EC_50_ (nM)
KI-184	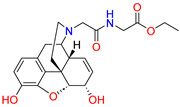	29.4 ± 4.4	6800
KI-186	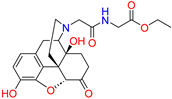	59.8 ± 3.8	13,644
KI-195	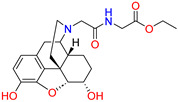	27.5 ± 4.8	8640
KI-209	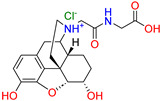	13.6 ± 1.2	395
KI-210	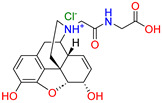	50.7 ± 0.5	5025
KI-211	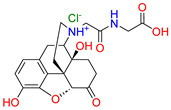	40.9 ± 4.1	10,406
KI-214	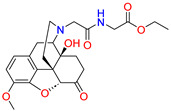	28.4 ± 6.8	16,507
KI-216	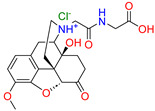	26.5 ± 3.3	4496

E_ma_x and EC_50_ values were calculated from isolated MVD experiments. Briefly, vasa deferentia were taken out from male NMRI mice (35–45 g) then cleaned from tissues and suspended between two electrodes in organ baths of 5 mL volume with 0.1 g initial tension. The organ baths were kept at 36 °C and filled with Krebs solution, of the following composition (mM/L): NaCl, 118.0; Na-HCO_3_, 25.0; KCl, 4.7; KH_2_PO_4_, 1.2; glucose, 11.0; and CaCl_2_, 2.5 aerated with carbogen (95% O_2_ + 5% CO_2_) and. The stimulation parameters were as follows: field stimulation, pairs (100 ms pulse distance) of rectangular impulses (1 ms pulse width, 9 V/cm, i.e., supramaximal intensity) were repeated every 10 s. The muscle contractions were monitored by LabChart 6.0 software. After equilibration time (20–30 min), the first dose of test compound was given, and concentration–effect curves were constructed in a cumulative manner [99]. Data were obtained from n = 4–7 animals per molecule.
